# COVID-19 vaccine induced poor neutralization titers for SARS-CoV-2 omicron variants in maternal and cord blood

**DOI:** 10.3389/fimmu.2023.1211558

**Published:** 2023-07-03

**Authors:** Sakthivel Govindaraj, Narayanaiah Cheedarla, Suneethamma Cheedarla, LesShon S. Irby, Andrew S. Neish, John D. Roback, Alicia K. Smith, Vijayakumar Velu

**Affiliations:** ^1^ Department of Pathology and Laboratory Medicine, Emory University School of Medicine, Atlanta, GA, United States; ^2^ Division of Microbiology and Immunology, Emory Vaccine Center, Emory University, Atlanta, GA, United States; ^3^ Department of Gynecology and Obstetrics, Emory University School of Medicine, Atlanta, GA, United States

**Keywords:** COVID-19 vaccination, pregnancy, cord blood, omicron, variants of concern

## Abstract

**Introduction:**

Maternally derived antibodies are crucial for neonatal immunity. Understanding the binding and cross-neutralization capacity of maternal and cord antibody responses to SARS-CoV-2 variants following COVID-19 vaccination in pregnancy can inform neonatal immunity.

**Methods:**

Here we characterized the binding and neutralizing antibody profile at delivery in 24 pregnant individuals following two doses of Moderna mRNA-1273 or Pfizer BNT162b2 vaccination. We analyzed for SARS-CoV-2 multivariant cross-neutralizing antibody levels for wildtype Wuhan, Delta, Omicron BA1, BA2, and BA4/BA5 variants. In addition, we evaluated the transplacental antibody transfer by profiling maternal and umbilical cord blood.

**Results:**

Our results reveal that the current COVID-19 vaccination induced significantly higher RBD-specific binding IgG titers in cord blood compared to maternal blood for both the Wuhan and Omicron BA1 strain. Interestingly, the binding IgG antibody levels for the Omicron BA1 strain were significantly lower when compared to the Wuhan strain in both maternal and cord blood. In contrast to the binding, the Omicron BA1, BA2, and BA4/5 specific neutralizing antibody levels were significantly lower compared to the Wuhan and Delta variants. It is interesting to note that the BA4/5 neutralizing capacity was not detected in either maternal or cord blood.

**Discussion:**

Our data suggest that the initial series of COVID-19 mRNA vaccines were immunogenic in pregnant women, and vaccine-elicited binding antibodies were detectable in cord blood at significantly higher levels for the Wuhan and Delta variants but not for the Omicron variants. Interestingly, the vaccination did not induce neutralizing antibodies for Omicron variants. These results provide novel insight into the impact of vaccination on maternal humoral immune response and transplacental antibody transfer for SARS-CoV-2 variants and support the need for bivalent boosters as new variants emerge.

## Introduction

Pregnant women are at higher risk during severe acute respiratory syndrome coronavirus 2 (SARS-CoV-2) infection, and its variants of concern (VOCs) increased severity in pregnant women (1–3). Vaccination is an effective preventive strategy and is recommended during pregnancy (3–6). Studies have demonstrated that pregnant women elicit a rapid antibody response to the COVID-19 vaccine and that anti-receptor binding domain (RBD) IgG and spike-specific neutralizing antibodies cross the placenta (7, 8). Studies have also found that both binding and neutralizing antibodies were higher in vaccinated pregnant women compared to natural infection (7, 8). SARS-CoV-2 variants remain and can lead to surges in infection rates, and understanding the immune response to VOCs during pregnancy is highly important. The Omicron subvariants BA1 and BA2 were the dominant variants of SARS-CoV-2 from July to November 2022 and displayed substantial neutralization escape as compared with previous variants (9–11). Additional Omicron variants have recently emerged, including the BA4/BA5 (12). Apart from the N501Y and D614G mutations, all of these Omicron variants (BA1, BA2, and BA4/BA5) shared unique mutations. Deletions of H69/V70 play a major role in the binding of ACE-2 receptors and increase infectivity by enhanced incorporation of the cleaved spike into the virions (13, 14). Interestingly, the BA2 variant gained back deletions of H69 and V70, which was shed by the BA1 and BA4/BA5 variants. Moreover, the BA2 and BA4/BA5 variants acquired the new mutations T19I, A27S, V213G, T376A, D405N, and R408S, which were not present in the BA1. BA1, BA2, and BA4/BA5 have rapidly increased in frequency of infection rate and confer neutralizing escape (15, 16). However, the ability of these variants to evade neutralizing antibodies induced by current vaccination during pregnancy is unclear. Here, we studied the profile of the maternal blood antibody response at delivery following COVID-19 vaccination and compared them with paired cord blood.

## Methods

### Study design and participants

Pregnant women and cord blood samples: The study was approved by the Institutional Review Board (IRB00101931) of Emory University. Per protocol, pregnant women were approached at Emory Hospital without any selection bias. Pregnant women who provided informed consent were eligible for the research blood draw. Blood samples were processed to isolate plasma and mononuclear cells. Pregnant women aged 18 years or older who spoke English or Spanish and were vaccinated against COVID-19 during pregnancy were offered enrollment into the Emory Prospective Opportunity for Women’s Health Research Initiative (EMPOWR). Patients who did not speak either English or Spanish as their first language were not approached. Demographic and health information, including maternal age, race/ethnicity, insurance status, and medical comorbidities such as obesity, chronic hypertension, and pre-gestational diabetes were abstracted from the medical record, as well as relevant maternal and birth outcomes. Clinical characteristics related to vaccination, specifically the type of vaccine, the timing of first and second doses, and the interval from vaccination to delivery, were collected prospectively. For this prospective cohort study, we selected pregnant women enrolled in EMPOWR who planned to deliver at Emory University Hospital Midtown in Atlanta, GA, USA, and who received both doses of the mRNA COVID-19 vaccines (Moderna mRNA-1273 or Pfizer BNT162b2) with their second dose at least 14 days prior to delivery. One participant reported having a history of COVID-19 in the past. All patients provided written informed consent in the study (n = 24) and delivered between January and August 2021. The demographic and clinical characteristics of the mother are displayed in **Table 1**. After enrollment, maternal and cord blood was collected into EDTA tubes during delivery admission. Blood samples were processed to obtain plasma and stored at −80°C until the antibodies were measured. Methods for plasma analysis for maternal and cord blood antibody concentrations of anti-RBD antibodies and neutralizing antibodies following vaccination have previously been published (5, 7).

**Table 1:** Demographic and clinical characteristics of a cohort of pregnant women who received COVID-19 vaccination^a^.

Characteristic	Value
Demographic	N = 24
Maternal age at delivery (years)	34 ± 3.8
Race and ethnicity	
Non-Hispanic White	17 (71)
Non-Hispanic Black	3 (12.5)
Asian	3 (12.5)
Hispanic any race	1 (4)
Pre-gravid BMI	
18–24.9	14 (58.5)
25–29.9	6 (25)
30.0–39.9	3 (12.5)
≥40	1 (4)
Medical comorbidities	
Obesity (BMI > 30)	4 (16.7)
Asthma	–
Gestational hypertension	4 (16.7)
Pregestational diabetes	4 (16.7)
Mental health	–
Previous SARS-CoV-2 infection	1 (4)
Others	3 (12.5)
mRNA vaccination	
Gestational age at vaccination (weeks)	26 (19–30)
Vaccine type	
Moderna	8 (33)
Pfizer	16 (67)
Timing of vaccine (1st dose) ^c^	
First trimester	0
Second trimester	8 (33.3)
Third trimester	15 (62.5)
Obstetric data	
Gestational age at delivery (weeks)**^b^**	39 ± 1
Mode of delivery	
Vaginal	16 (67)
Cesarean	8 (33)
Preterm birth, gestational age < 37 weeks	–
Infant birth weight	3,257 ± 408

BMI, body mass index (calculated as weight in kilograms divided by the square of height in meters); ^a^ Data are presented as mean ± standard deviation, median (interquartile range), or number (percentage). ^b^ Refers to gestational age at receipt of the first dose. ^c^ One participant does not have a date for the first dose of vaccination.

### RBD binding assay

SARS CoV-2 Wuhan-WT and Omicron BA1 RBD proteins were expressed in Expi293 cells and purified by affinity column chromatography followed by size exclusion chromatography (SEC) and used for detection of RBD binding antibodies; briefly, SARS-CoV-2 RBD (Wuhan and Omicron BA1) was coated on Corning Costar high binding assay plates at a concentration of 0.5 μg/ml in 100 μl phosphate-buffered saline (PBS) at 4°C overnight. Plates were blocked for 1 h at room temperature in PBS/0.2%Tween/1% BSA (ELISA buffer). Plasma samples were aliquoted and stored at −80°C before use. Samples were serially diluted 1:4 in ELISA buffer starting at a dilution of 1:50 (1:50 to 1:328,050). One hundred microliters of each dilution was added and incubated for 1 h at room temperature. One hundred microliters of horseradish peroxidase-conjugated anti-human IgG (Jackson ImmunoResearch, Catalog # 109-035-088) diluted 1:4,000 in ELISA buffer was added and incubated for 1 h at room temperature. Development was performed using o-phenylenediamine substrate (Sigma) in 0.05 M phosphate-citrate buffer, pH 5.0, supplemented with 0.012% hydrogen peroxide before use. Reactions were stopped with 1 M HCl and absorbance was measured at 490 nm. Between each step, samples were washed three times with 200 μl of PBS-0.5% Tween 20. Endpoint titers were calculated through a dose–response curve fit with non-linear regression using the software Graphpad Prism 9.0.

### Pseudovirus neutralization assay

Neutralization activities against SARS-CoV-2 WT (Wuh-1), Delta (B.1.617.2), Omicron BA1, BA2, and BA4/BA5 strains were measured in a single round of infection assay with pseudoviruses (17, 18). Briefly, to produce SARS-CoV-2 WT, Delta, and Omicron (BA1, BA2, and BA4/BA5) pseudoviruses, an expression plasmid bearing codon-optimized SARS-CoV-2 full-length S plasmid (parental sequence Wuhan-1, Genbank #: MN908947.3) was co-transfected into HEK293T cells (ATCC#CRL-11268) with plasmids encoding non-surface proteins for lentivirus production and a lentiviral backbone plasmid expressing a Luciferase-IRES-ZsGreen reporter, HIV-1 Tat, and Rev packing plasmids (BEI Resources NR-53818) and pseudoviruses harvested after 48 h of post-transfection, and titration was performed. Pseudoviruses were mixed with serial dilutions (1:50 to 1:328,050) of plasma, incubated for an hour for the reaction to happen at a 37°C, 5% CO_2_ incubator, and then added to monolayers of ACE-2-overexpressing 293T cells (BEI Resources NR-52511), in duplicate. Forty-eight hours after infection, cells were lysed, luciferase was activated with the Luciferase Assay System (Bright-Glo Promega), and relative light units (RLU) were measured on a synergy Biotek reader. After subtraction of background RLU (uninfected cells), % neutralization was calculated as 100× [(virus only control)−(virus plus antibody)]/(virus only control). Dose–response curves were generated with a four-parameter nonlinear function, and titers were reported as the serum dilution or antibody concentration required to achieve 50% (50% inhibitory dilution [ID_50_]) neutralization. Statistical analysis was performed using the software GraphPad prism 9.0 for the determination of ID_50_ values through a dose–response curve fit with non-linear regression.

### Statistical analysis

Mann–Whitney test and paired t-tests were used for comparisons of RBD-specific binding titer, neutralization titer, and transfer ratio between the cord and maternal blood. Correlation between the assays was performed by the Pearson r correlation method and linear regression analysis. All statistical analysis was performed using GraphPad PRISM version 9 (San Diego, CA), and SPSS package version 27.0 (Chicago, IL). Each specimen was tested in duplicate. All statistical tests were two-sided unless otherwise noted, and statistical significance was assessed at the 0.05 level; the *p* values for detectable antibodies in the maternal and cord blood were determined using Fisher’s exact test.

## Results

**Figure 1:**
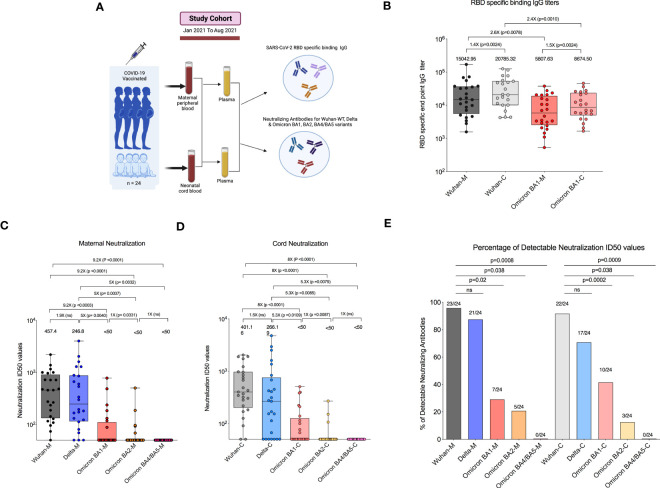
COVID-19 mRNA vaccination in pregnant women lacks neutralizing antibodies to Omicron subvariants. **(A)** Experimental design—Recruitment of COVID-19-vaccinated pregnant women who had received a vaccination 14 days before delivery were enrolled in the study. **(B)** Box plot represents RBD-specific end point IgG titers for Wuhan (black) and Omicron BA1 (red) variants in both maternal and cord blood. **(C)** Box plot represents neutralizing antibody titers for maternal blood samples by luciferase-based pseudovirus neutralization assay for Wuhan (black), Delta (blue), Omicron BA1 (red), Omicron BA2 (orange), and Omicron BA4/BA5 (pink) variants. **(D)** Neutralizing antibody titers for cord blood samples for Wuhan (gray), Delta (light blue), Omicron BA1 (light red), Omicron BA2 (light orange), and Omicron BA4/BA5 (light pink) variants. **(E)** The percentage of detectable neutralization antibody titers in the maternal and cord blood for Wuhan, Delta, and Omicron subvariants. ns, Non Significant.

Twenty-four COVID-19-vaccinated pregnant women were enrolled and provided paired maternal and cord blood at delivery (**Figure 1A**). We first assessed the RBD-specific binding IgG levels specific to the Wuhan-WT and Omicron BA1 variants in participants who had been vaccinated with two doses of COVID-19 mRNA vaccines. After the second dose, the RBD-specific binding IgG titers to the Wuhan-WT-WA1/2020 strain had a median endpoint titer of 15,042 in the maternal blood, whereas the Omicron BA1 variant showed 2.6-fold lower binding with a median titer of 5,807. Similarly, the median binding titer was 20,785 for the Wuhan-WT in the cord blood, and the median binding titer for the Omicron BA1 variant was 8,674, which was 2.4-fold lower (**Figure 1B**).

**Figure 2:**
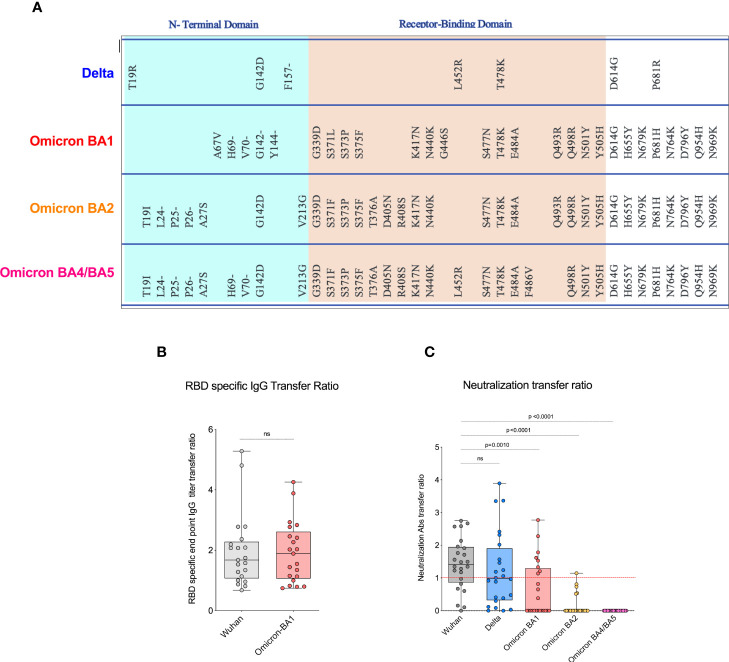
COVID-19 mRNA vaccines induced significantly lower neutralizing antibody transfer ratio. **(A)**. Shows spike protein sequences comparing Delta, and Omicron BA1, BA2, and BA4/BA5 subvariants; mutations as compared with WA1/2020 spike protein are shown. **(B)**. Displays the RBD-specific end point IgG titer transfer ratio for Wuhan and Omicron BA1 variant. The transplacental transfer ratio was calculated as cold blood IgG concentration divided by maternal IgG concentration. **(C)**. The data shows the neutralization antibody transfer ratio for Wuhan, Delta, and Omicron variants, and the transfer ratio was poor in Omicron variants. ns, Non Significant.

We next evaluated neutralizing antibody titers in all 24 participants for Wuhan-WT, Delta, and Omicron subvariants BA1, BA2, and BA4/5 as previously described (18). The neutralizing antibody titers to the Wuhan-WT and Delta variants were comparable, but they are significantly higher than the Omicron subvariants in the maternal blood. The median neutralizing antibody titer to the Wuhan-WT was 457 while the titer to the Delta was 246, and all the Omicron subvariants BA1, BA2, and BA4/BA5 were <50 (**Figure 1C**) in the maternal blood. Similarly, the neutralizing antibody titers to the Wuhan-WT and Delta variants were higher than the Omicron subvariants in the cord blood. The median neutralizing antibody titer to the Wuhan-WT was 401, while the titer to the Delta was 266, and all the Omicron subvariants BA1, BA2, and BA4/BA5 were <50 (**Figure 1D**) in the cord blood. There was no difference observed between maternal and cord blood-neutralizing antibody titers for all the variants (**Supplemental Figure 1**). In line with that, the percentage of detectable neutralizing antibodies for Omicron subvariants are significantly dropped compared to the Wuhan-WT and Delta variant (**Figure 1E**). These data suggest that the Omicron variants escape neutralization because of multiple mutations in the N terminal and RBD domain compared to wild type (16, 19, 20) (**Figure 2A**).

**Figure 3:**
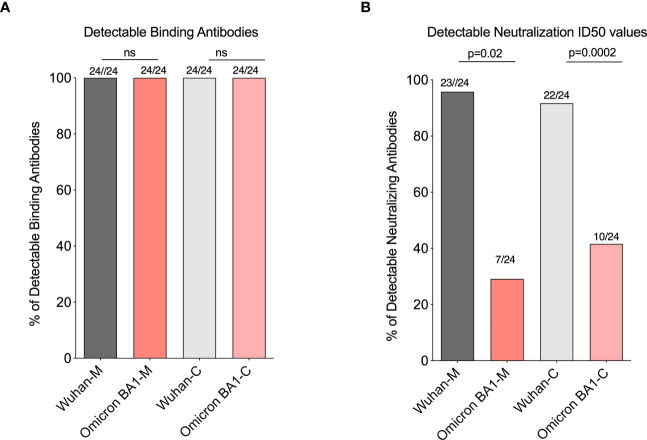
A significant drop in the percentages of seropositive individuals with detectable neutralizing antibodies for the omicron BA1 variant. **(A)** Box plots represent similar detectable RBD-specific binding IgG antibodies in the maternal and cord blood for Wuhan and Omicron BA1 variants. **(B)** Box plot represents a significant drop in the detectable neutralizing antibodies level with the Omicron BA1 variant compared to wild type in the maternal and cord blood. ns, Non Significant.

**Figure 4:**
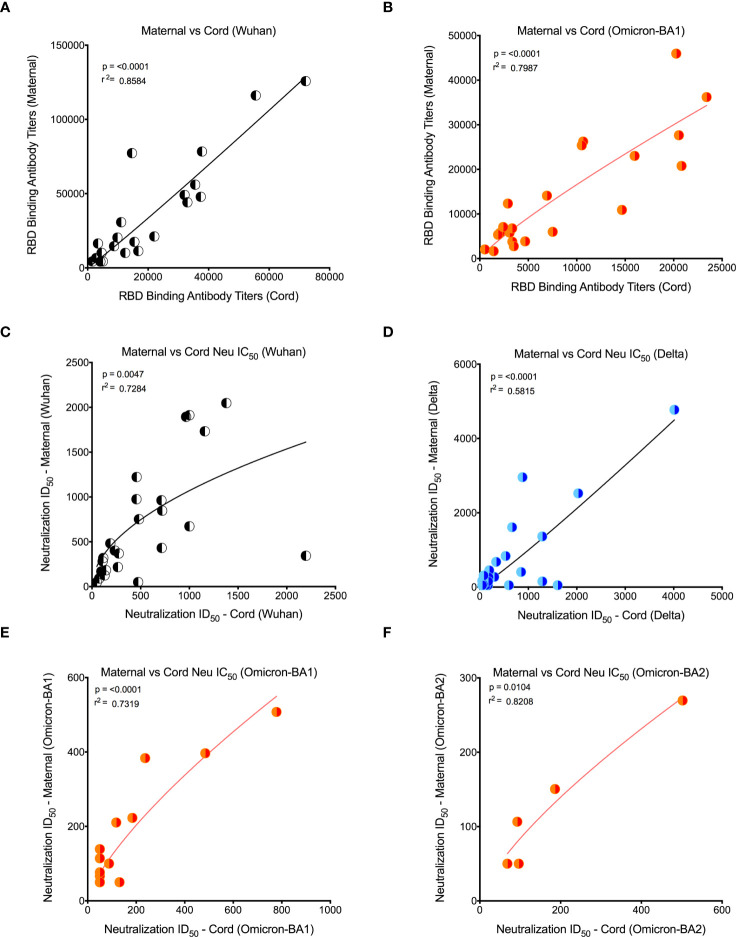
Detectable binding and neutralizing antibody levels in maternal and cord blood correlated positively for all variants. **(A)** Wuhan RBD-specific binding IgG antibody levels correlation between maternal and cord blood. **(B)** Omicron RBD-specific binding antibody level correlation between maternal and cord blood. **(C)** Wuhan neutralizing antibody level correlation between maternal and cord blood. **(D)** Delta-specific neutralizing antibody level correlation between maternal and cord blood. **(E)** Omicron BA1-specific neutralizing antibody level correlation between maternal and cord blood. **(F)** Omicron BA2-specific neutralizing antibody level correlation between maternal and cord blood. Note that for all Omicron variants, only detectable antibody levels are plotted.

Our recent study in pregnant women has shown higher IgG transfer ratios in pregnant vaccinated women *vs*. those with natural SARS-CoV-2 infection (7). Hence, first we calculated the placental transfer ratio of RBD-specific binding IgG antibodies for the Omicron variant compared to the Wuhan-WT, but it was not different (**Figure 2B**). We then calculated the placental transfer ratio of neutralization titers across all Wuhan-WT, Delta, and Omicron subvariants BA1, BA2, and BA4/BA5. The neutralization transfer ratio was significantly lower for Omicron BA1 (p = 0.001), BA2 (p < 0.0001), and BA4/BA5 (p < 0.0001) variants when compared to the Wuhan-WT (**Figure 2C**). These data suggest that although there is a comparable binding antibody detection between Wuhan-WT and Omicron BA1, there is a significant drop in the percentage of seropositive individuals with detectable neutralizing antibodies as demonstrated in **Figures 3A, B**. We then looked at the association of detectable binding and neutralizing antibody titers between maternal and cord blood. The detectable binding and neutralizing antibodies correlate with maternal and cord blood respectively for all variants (**Figure 4**). First, the maternal binding antibody levels in Wuhan and Omicron BA1 correlated positively with cord blood binding antibody levels (**Figures 4A, B**). Then, we performed correlation analysis with neutralizing antibody titers, similar to the binding antibodies, and the detectable neutralizing antibody levels correlate positively with respective cord blood across all variants (**Figures 4B–D**, **Supplemental Figure 2**). Overall, these data suggest that wherever you have detectable binding and neutralizing antibodies in maternal blood, you tend to have detectable antibodies in the cord blood, suggesting that boosting protective antibody levels in maternal will help infant immunity.

## Discussion

In this prospective study, we profiled the antibody response from a cohort of patients vaccinated with two doses of a COVID-19 vaccine during pregnancy and then compared the data to the immune response between maternal and cord blood. Similar to previously published studies, our results demonstrate that two doses of mRNA COVID-19 vaccination during pregnancy was able to generate a robust IgG response, with almost all maternal and cord samples demonstrating detectable anti-RBD-IgG antibodies (21). The vaccine is equally effective in eliciting an antibody response in individuals as it is in the general population (22). However, unlike the natural infection (5, 7, 8), when the IgG antibody titers were compared, the cord blood induced higher levels of antibody for both wild-type Wuhan and Omicron, respectively, compared to paired maternal samples. In natural infection, the IgG antibody titers are lower in cord blood compared to maternal blood (5, 7, 8). Interestingly, in the vaccinated cohort, we saw that for binding RBD-specific IgG, the median titer was higher in cord blood samples compared to maternal, but the neutralization was comparable between maternal and cord blood.

This study has notable strengths. By design, it compares the antibody responses for variants of concern in pregnant individuals with proof of receipt of both doses of the COVID-19 vaccine greater than 14 days before delivery and compares them with variants of concern. This is the first study to assess the neutralizing antibody response to Wuhan-WT and compared them with variants of concern in cord blood and in paired maternal blood. At the time of writing, this study was the only research, to our knowledge, to assess the antibody response to SARS-CoV-2 variants in pregnant women’s maternal and cord blood samples, comparing paired maternal blood neutralizing antibody response across Wuhan-WT, Delta, and Omicron subvariants BA1, BA2, and BA4/BA5. Our main finding was that COVID-19-vaccinated pregnant women lack neutralizing capacity significantly to the Omicron subvariants BA1, BA2, and BA4/BA5 compared to the Wuhan-WT and Delta variants. Furthermore, recent studies in the general population have shown significant neutralizing escape for SARS-CoV-2 Omicron variants in the vaccinated cohort. This finding is consistent with the results of the present study in pregnant women (9, 12, 23). Our study had a few limitations; first, it was a single-center (Atlanta- cohort) study with a small sample size. Since we had a small number of cord and maternal samples with few numbers of pregnant women who had detectable neutralizing antibodies for the Omicron variants, we accounted for this by analyzing the titers in two ways; first, by looking at all samples in aggregate and then by removing the samples with non-detectable levels. In addition, we have not analyzed the data concerning the trimester, which would require more samples.

Our main finding was that COVID-19-vaccinated pregnant women lack neutralizing capacity significantly to the Omicron subvariants BA1, BA2, and BA4/BA5 compared to the Wuhan-WT and Delta variant. Our data show that the Omicron subvariants BA1, BA2, and BA4/BA5 escaped neutralizing antibodies substantially more effectively than the Wuhan-WT and Delta variants. The neutralizing antibody titers to BA1, BA2, and BA4/BA5 were significantly lower than titers to the Wuhan-WT and Delta strains in this cohort. These findings suggest that the Omicron variants may reduce the efficacy of current mRNA vaccines. As per the World Health Organization COVID-19 data (https://covid19.who.int/), BA1, BA2, and BA4/BA5 variants increased the numbers of COVID-19 infections worldwide relative to the Alpha (UK strain-B1.1.7) and Delta (Indian, B1.617.2) strains. The incorporation of multiple new SARS-CoV-2 variant antigens in bivalent vaccines would be greatly beneficial for pregnant women to promote infant immunity. In order to achieve effective protection against the highly prevalent SARS-CoV-2 Omicron variants, it is crucial to incorporate both the ancestral and Omicron strains of the virus into the COVID-19 vaccine. To prevent adverse outcomes associated with SARS-CoV-2 infection during pregnancy, it is recommended that pregnant women are updated with the recommended COVID-19 vaccines and, if available, receive bivalent mRNA booster when eligible (24, 25).

## Data availability statement

The original contributions presented in the study are included in the article/**Supplementary Material**. Further inquiries can be directed to the corresponding authors.

## Ethics statement

The studies involving human participants were reviewed and approved by Institutional Review Board (IRB00101931) of Emory University. The patients/participants provided their written informed consent to participate in this study.

## Author contributions

VV and AS conceived the study. VV, SG, and NC developed the methodology. SG, VV, NC, SC, and LI conducted the investigations. SG, NC, and VV wrote the original draft. VV, AS, AN, JR, and NC reviewed and edited the final manuscript. VV acquired funding; and VV, AS, AN, and JR supervised the study. All authors contributed to the article and approved the submitted version.
