# Supplementary material for: COVID-19 vaccine induced poor neutralization titers for SARS-CoV-2 omicron variants in maternal and cord blood
Source: Front Immunol. 2023 Jul 3;14:1211558. doi: 10.3389/fimmu.2023.1211558 (PMC10350671; doi:10.3389/fimmu.2023.1211558)
Supplement: Supplementary file 1 [file DataSheet_1.pdf]

Supplemental Information

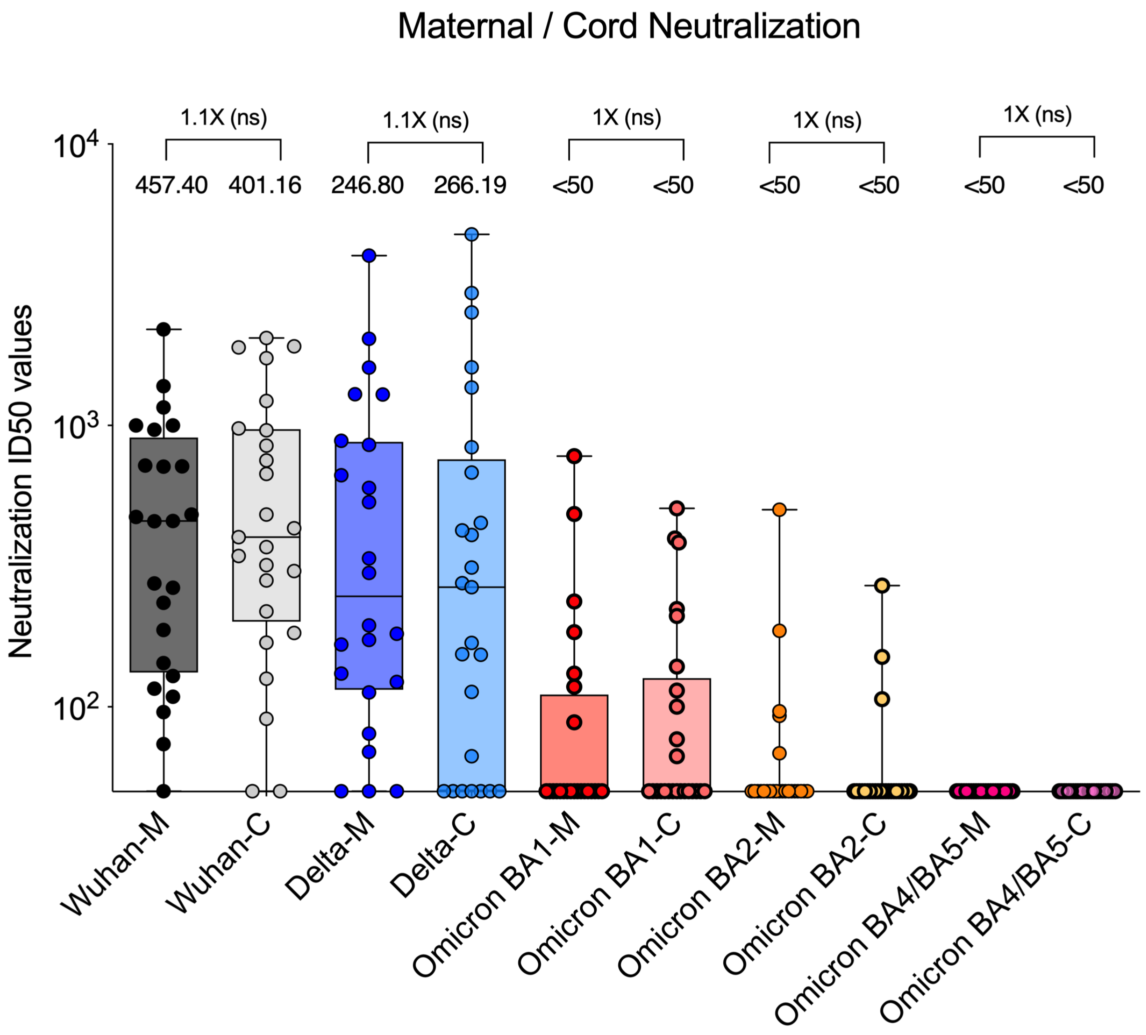

Supplemental Figure 1. Maternal and Cord blood lacks neutralizing antibodies to Omicron subvariants. Box plots represent the comparison of neutralization antibody titers between maternal and cord blood for Wuhan, Delta, and Omicron BA1, BA2, and BA4/BA5 subvariants.

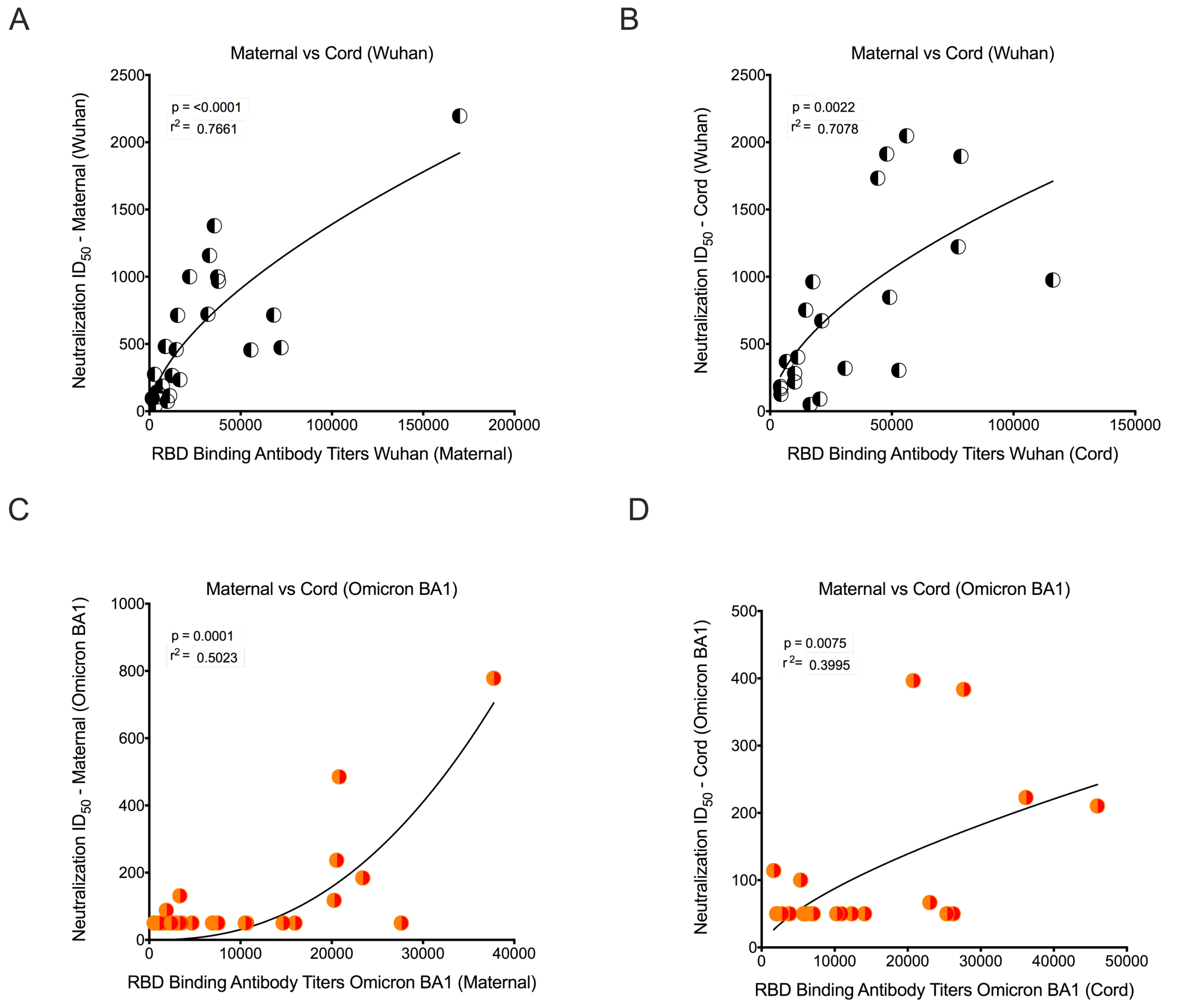

Supplemental Figure 2. Antibody levels in Maternal and Cord blood correlated positively for all Wuhan and Omicron BA1 variant. (A). Shows Wuhan neutralizing antibody levels correlation between maternal and **(B)** cord blood. **(C).** Shows Omicron BA1 specific neutralizing antibody levels correlation between maternal and **(D)** cord blood. Note that for Omicron BA1 variant both detectable and undetectable antibody levels are plotted.
